# United-Atom Molecular Dynamics Study of the Mechanical and Thermomechanical Properties of an Industrial Epoxy

**DOI:** 10.3390/polym13193443

**Published:** 2021-10-08

**Authors:** Riki Maicas, Irena Yungerman, Yarden B. Weber, Simcha Srebnik

**Affiliations:** 1 Department of Chemical Engineering, Technion-Israel Institute of Technology, Haifa 32000, Israel; rikimai@gmail.com (R.M.); yu.irena@gmail.com (I.Y.); 2 Rafael—Advanced Defense Technologies, Haifa 32000, Israel; yardenv@rafael.co.il; 3 Department of Chemical and Biological Engineering, University of British Columbia, Vancouver, BC V6T1Z3, Canada

**Keywords:** epoxy, molecular dynamics, mechanical deformation

## Abstract

Epoxy resins are the most commonly used adhesives in industry due to their versatility, low cost, low toxicity, low shrinkage, high strength, resistance to moisture, and effective electrical resistance. These diverse properties can be tailored based on the chemical structure of the curing agent and the conditions of the curing process. Molecular simulations of epoxy resins have gained attention in recent years as a means to navigate the vast choice of chemical agents and conditions that will give the required properties of the resin. This work examines the statistical uncertainty in predicting thermodynamic and mechanical properties of an industrial epoxy resin using united atom molecular dynamics simulation. The results are compared with experimental measurements of the elastic modulus, Poisson’s ratio, and the glass transition temperature obtained at different temperatures and degrees of curing. The decreasing trend of the elastic modulus with increasing temperature is accurately captured by the simulated model, though the uncertainty in the calculated average is large. The glass transition temperature is expectedly overpredicted due to the high rates accessible to molecular simulations. We find that Poisson’s ratio is particularly sensitive to sample anisotropy as well as the method of evaluation, which explains the lack of consistent trends previously observed with molecular simulation at different degrees of crosslinking and temperatures.

## 1. Introduction

The versatility, low cost, low toxicity, low shrinkage, high strength, resistance to moisture, and effective electrical resistance of epoxy-based adhesives lends them well to a variety of industries. This flexible yet tough adhesive offers tunable mechanical and thermomechanical properties based on the choice of curing agent (e.g., amines, phenols, mercaptans, isocyanates, or acids) and the conditions of the curing process [1,2], as well as the kinetics of the crosslinking reaction [3,4]. Ultimately, it is molecular scale events that determine the material behavior, where details such as local chain stiffening can significantly affect mechanical response [5]. Such nanoscale response to mechanical deformation can in principle be probed with molecular simulation. However, proper measurements require significant computational resources, particularly because of the non-ergodic nature of the chemically crosslinked system. Nonetheless, with the increase in computational power, more research is being devoted to the development of molecular models of epoxy and prediction of their mechanical and thermomechanical properties. Today, molecular dynamics presents the only practical tool for molecular modeling of time-dependent material properties with sufficient lengthscales that can capture the crosslinked nature of the polymer resin.

Atomistic molecular dynamics simulations have primarily focused on diglycidyl ether bisphenol A (DGEBA)-based epoxies with a variety of amine-based curing agents. In one of the first studies of these systems, Yarovsky and Evans [6] established the crosslinking method based on distance constraints. This simple crosslinking model was able to capture the expected shrinkage of the resin upon curing. While modeling realistic crosslinking reactions requires a reactive force field that allows formation and breaking of chemical bonds, reactive models are not yet computationally feasible for epoxy due to the long reaction timescales relative to computational timescales [7]. Fortunately, proper equilibration between crosslinking events based on distance constraints allows for efficient simulations with a resulting distribution of primary, secondary, and tertiary amine groups that are in fair agreement with experiments [8]. Though local structure formation might occur, particularly at high degrees of curing [9], the distribution of crosslinked sites was found to negligibly affect the glass transition temperature and linear expansion coefficient of epoxy [10], providing validity for such *ad hoc* methods. Adding energy minimization prior to the reaction allowed for higher degrees of crosslinking to be achieved more efficiently and resulted in fair agreement with experimental measures of elastic constants and Poisson’s ratio [11]. Later studies of similar systems and with similar reaction schemes showed fair agreement with experimental measurements of mechanical and thermal properties, including estimation of the glass transition temperature [11,12,13,14,15,16,17,18], regardless of the crosslinking method and choice of force field. Although a direct comparison of force fields revealed non-negligible force-field dependence of predicted thermodynamic and mechanical properties [12,13], most of the measures from all force fields fell within the reported experimental data.

The bulk modulus is an important measure of adhesive performance, such as in the design of adhesive joints, where deformation of the adhesive can lead to premature failure of the joint. The bulk modulus can be readily calculated from simulations either from volumetric fluctuations at constant pressure or from isotropic compression. While prediction of the bulk modulus and other elastic properties using dynamic simulations of DEGBA/DETDA were shown to agree with the theory of linear elasticity [18], the bulk modulus obtained from simulations was found to be consistently higher than experimental measures. This may be explained by the absence of defects in the simulated systems [13]. However, Bandyopadhyay et al. [15] observed a weakly decreasing trend of the bulk modulus with increasing crosslinking density, counter to expectation, suggesting that volumetric deformation may not be a sufficiently sensitive measure at high degrees of crosslinking. In addition to the small and idealized sample size, these differences between simulations and experiments may also stem from the orders of magnitude difference in modeled deformation rates compared with experimental conditions, which leads to vastly differing stress-strain curves [7].

The transition from the glassy to rubbery state is modulated by various modes of relaxation that are defined by different scales of molecular motion. Below the glass transition temperature (*T_g_*), relaxation is limited to local molecular motion that occurs at relatively small timescales. Above *T_g_*, larger parts of the polymer chain begin to fluctuate and rotate. The transition itself depends on the timescale of the experiment [19]. The significantly higher cooling (or heating) rates used in simulation limit the relaxation of the chains to local motion and hence result in consistently higher predictions of *T_g_* [16,20]. In spite of the vast differences in deformation rates accessible to molecular simulation, results from many reported computational studies suggest that material properties can be predicted and the elastic response of the material can be correlated to experimental measurements with surprising accuracy.

Both experiments and computations provide a range of results for many mechanical and thermomechanical properties for any given epoxy system. This wide variance in measured and predicted behavior stems from differences in the curing process and the large scatter in simulation measurements inherent to systems with nanoscale dimensions. Additionally, the method of property evaluation from the raw data can lead to vastly different predictions. For example, the elastic modulus obtained from molecular models invariably strongly depends on the range of deformation from which it is extracted [14]. This stresses the fact that measurements obtained from molecular simulations must involve a number of independent samples so that average properties can be compared consistently with experiments.

In this work, we address the expected deviation in simulated quantities of an epoxy sample, including the elastic, bulk modulus, Poisson’s ratio, and glass transition temperature. Predictions from the simulations are compared with experimental measurements for an industrial epoxy used for adhesive joints. The united atom force field is used to model an epoxy resin based on EPON 815c cured with Epikure 3140. The complex chemical composition of the experimental system is modeled to minimize discrepancies between the simulated and experimental systems. Mechanical properties are calculated for a range of temperatures as well as different degrees of curing. To examine the spread of properties obtained from the simulations, mechanical tests are performed for five systems that have been independently crosslinked and are averaged over the three box axes when applicable. We discuss the average measurements obtained from the simulation and their distribution across the different samples for the evaluated properties.

## 2. Experimental Methods

Amine-cured epoxy specimens were prepared at a weight ratio of 2:1 from EPON 815C epoxy-based resin and Epikure 3140 amine curing agent, obtained from Hexion Inc., Columbus, OH, USA. EPON 815C is a bisphenol A-based epoxy of low viscosity and great wetting characteristics which consists of a mixture of 13.6% butyl glycidyl ether (BGE) and 86.4% bisphenol A diglycidyl ether (DGEBA). BGE is a reactive diluent that is often added to epoxy resins to lower their viscosity without compromising mechanical properties. Epikure^TM^ 3140 is a curing agent based on dimerized fatty acids and polyamines. It imparts high resistance to corrosion and permeation of moisture, as well as great adhesion properties. It consists of 16% triethylenetetramine (TETA) and 84% condensation products of polyamines and fatty acid C18 dimers. The dimerized fatty acid byproduct is a 36-carbons molecule derived from the conversion of unsaturated fatty acids. It is incorporated in the backbone of the curing agent to increase its flexibility and toughness.

Tensile specimens were prepared by mixing, degassing, and injecting the resin into molds to form dumbbell tension specimens, with a length of 115 mm and a neck cross section of 12.7 mm over 6.4 mm according to ASTM D638 [21]. The specimens were cured at room temperature for seven days and aged at room temperature for one year. Tensile tests were conducted at temperatures of −5 °C, room temperature (RT), and 60 °C. A deformation rate of 2 mm/min was applied with an Instron testing machine equipped with a video extensometer for simultaneous measurement of Poisson’s ratio. The glass transition temperature, *T_g_*, was measured using a DSC Q10 TA instruments analyzer. Two consecutive heating cycles were conducted to eliminate thermal history. The thermal expansion coefficient was measured below *T_g_* by a TMA Q400 TA analyzer. The DSC and TMA were purchased from TA Instruments, New Castle, DE, USA.

## 3. Molecular Model

**Figure 1:**
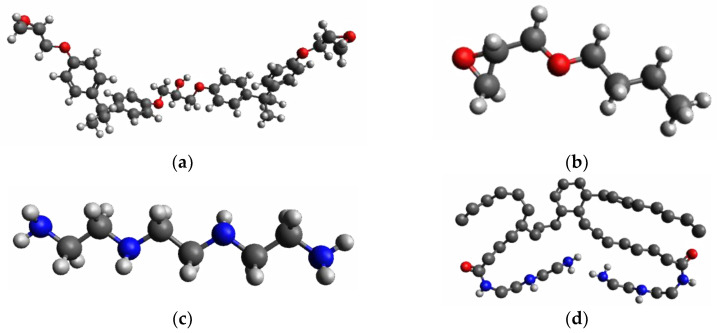
United atom depiction of the modeled EPON 815C/Epikure 3140 epoxy resin consisting of (**a**) DGEBA, (**b**) BGE, (**c**) TETA, and (**d**) dimerized fatty acid. Nonpolar hydrogens are implicitly included in the bonded carbon atom in the united atom model.

We investigate an epoxy system composed of EPON 815C epoxy and Epikure^TM^ 3140 curing agent, with similar chemical composition of the experimental resin. The chemical structure of the different components making up the resin is shown in Figure 1. The initial molecular structures were obtained using Avogadro open-source molecular builder and visualization tool (Version 1.2.0) [21].

Simulations were performed using LAMMPS [22] molecular dynamic (MD) simulator with the OPLS united atom force field [23]. Equilibration of the initial non-crosslinked blend (consisting of 4196 atoms with 45:34:11:20 molecular ratio of DGEBA:BGE:TETA:C18) was done under isothermal-isobaric conditions (NPT) with periodic boundaries and an integration timestep of 1 fs. An average density of 1.13 g/cm^3^ was obtained, which agrees well with experimental densities.

**Figure 2:**
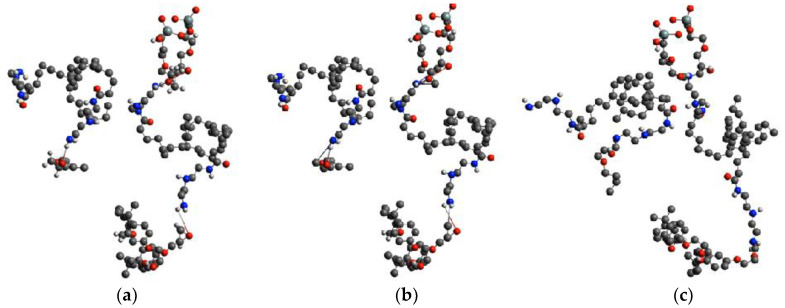
Simulation snapshots showing (**a**) crosslinking reaction between O (red) and H (white) atoms to from new O–H bond and (**b**) between C (grey) and N (blue) to form a new C–N bond, while amine N–H bond and epoxy C–O bonds are broken, and (**c**) the re-equilibrated system.

**Figure 3:**
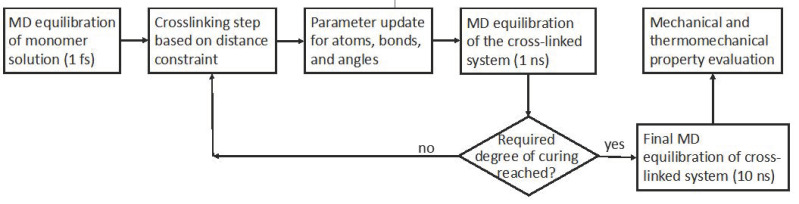
Flow diagram for the molecular dynamics and crosslinking simulation procedure.

Crosslinking was performed using a distance-based in-house algorithm combined with MD equilibration. Crosslinking sites were identified when either N/C or O/H atom pairs were found at a distance of 4.6 Å. Figure 2 shows a snapshot of the crosslinking process during the simulation. Following bond formation and breakage, potential parameters were updated and the system re-equilibrated before further crosslinking. The crosslinking reaction terminated when the desired degree of crosslinking was achieved. Equilibration of the final crosslinked system was carried out for an additional 10^7^ time steps (10 ns). The flow diagram of the simulation is given in Figure 3.

**Figure 4:**
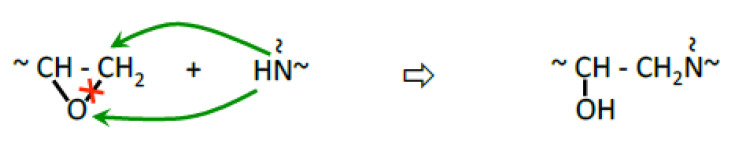
Schematic depiction of the crosslinking reaction between an epoxy group and an amine group, involving N–C and O–H bond formation between, and C–O and N-H bond breakage. The epoxy oxygen transforms into an alcohol group and the epoxy carbon transforms into a linear alkane carbon.

During the crosslinking reaction, the chemical identity of the participating atoms changes. Namely, the epoxy oxygen transforms into an alcohol and the epoxy carbon becomes a linear alkane carbon, as is depicted in Figure 4. Additionally, the topology of the evolving crosslinked molecule changes, and new partial charges must be defined as well as new potential parameters for the bonds, angles, and dihedrals.

Simulations were carried out for five independently crosslinked configurations. Tensile, compression, and thermal deformations were performed to evaluate mechanical and thermomechanical properties of the system at different temperatures below and above *T_g_*. The effective heating and cooling rate for the thermal deformation simulations was 25° K/ns, where samples were equilibrated for 0.1 ns after each temperature step change. Mechanical deformations were performed at constant engineering strain rates of 10^7^ s^−1^ and 10^9^ s^−1^.

## 4. Results and Discussion

### 4.1. Experimental

Experimental measurements of EPON 815C epoxy cured with Epikure 3140 at a weight ratio of 2:1 are presented in Table 1. The glass transition temperature for the samples was measured after 7 days of curing and after 1 year of aging, both at room temperature. Results are given in Table 2. The corresponding thermal expansion coefficient measured below the *T_g_* was 72 ppm/°C. The experimental measurements present averages over three independent specimens.

An order of magnitude drop in the tensile modulus is observed between measurements taken below the glass transition temperature and those taken just above *T_g_*, with a corresponding increase in Poisson’s ratio of nearly 20%. Moreover, the glass transition temperature is seen to be sensitive to the number of heating cycles. Aging raises the glass transition temperature significantly—by more than 10 °C. We focus our computational analysis on three of these measurements: elastic modulus, Poisson’s ratio, and the glass transition temperature.

### 4.2. Computational

We simulate the experimentally-tested epoxy adhesive with a 2:1 w/w mixture of EPON 815c and Epikure 3140. EPON 815c is a mixture of 86.4% DGEBA and 13.6 BGE. Epikure 3140 consists of 16% TETA and 84% condensation products of polyamines and fatty acid C18 dimers. This composition results in a significant excess of amine groups compared with epoxy groups such that side reactions can be assumed to be negligible [1]. The OPLS force field was checked for consistency with experimental measurements of the density of each of the individual components and some mixtures, where available. The results are shown in Table 3.

**Figure 5:**
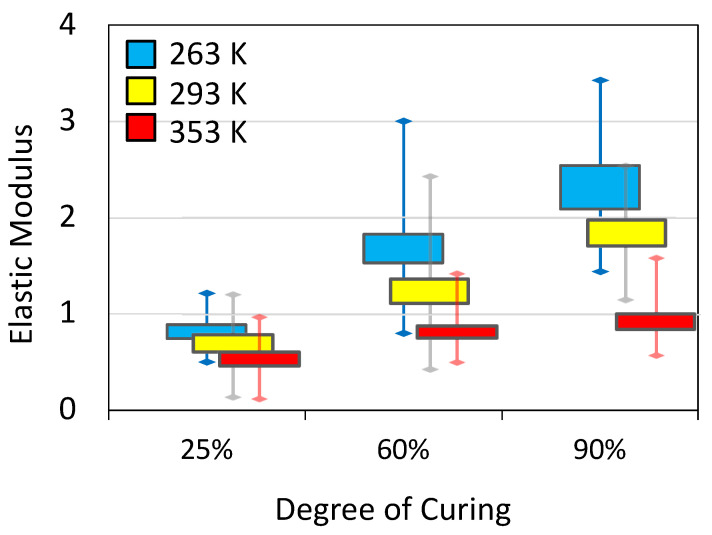
Box plots showing the dependence of Young’s modulus on the degree of crosslinking and temperature.

Simulations of mechanical and thermal deformations were compared with experimental measurements for five samples with various degrees of curing. Young’s modulus *E* was calculated from the slope of stress-stain curves in the elastic region during tensile deformation. We simulated tensile deformation of up to 5% using a constant strain rate, applied at engineering strain rates in the range 10^−7^–10^−9^ fs^−1^. These rates are equivalent to loads in the range of 10^6^–10^8^ s^−1^. Strain was applied independently to each of the three axes of the five individual samples for various temperatures and degrees of crosslinking. The values of *E* provided in Figure 5 present an average over 15 measurements for each temperature and degrees of crosslinking. *E* is seen to clearly depend on both temperature and the degree of crosslinking with the expected behavior. However, the average value from the simulation (taken at 90% degree of crosslinking) underestimates *E* at room temperature and above, but overestimates *E* at 263 K. As the temperature increases and the substance expands, the force holding the atoms together weakens. As a result, it is expected that the Young’s modulus will decrease proportionally. Overestimation of *E* is anticipated since the high deformation rates simulate an effectively lower temperature [24]. Since *E* was found to be independent of the local distribution of crosslinks [10], we attribute the underestimation of *E* at high *T* to the relatively homogeneous density of the simulated sample [25]. Notably, the distribution of *E* is wide for each set of measurements and results in significant overlap between the various conditions. Nonetheless, most measured values based on a single simulated deformation of a sample were within the simulated range [26] and experimental range, e.g., [27], of similar systems.

**Figure 6:**
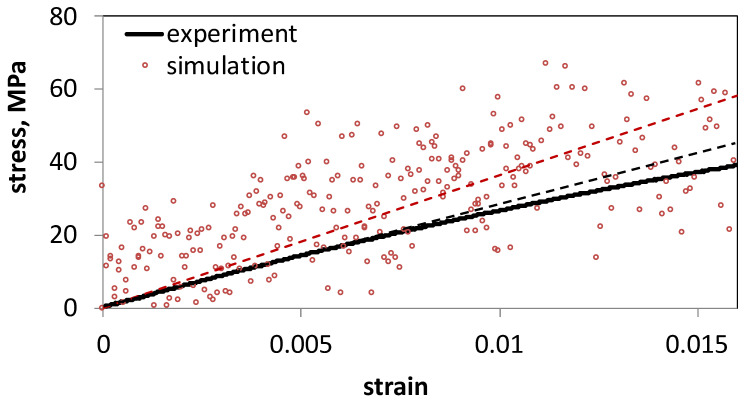
Stress versus strain for experimental measurements taken at room temperature and computational measurements at 25 °C and 90% cross-linking. Linear fits to the experimental data and simulation data are shown in black and red dashed lines, respectively.

The wide distribution of values seen in Figure 5 for all measurements reveals that the sensitivity for each simulation is large. Moreover, the distribution as well as average values are quite dependent on the range of deformation from which it is extracted ^11^. Extracting *E* from 2% strain versus 5% strain results in more than 20% difference in the average property (e.g., for 90% crosslinking at 293 K *E*_2%_ *=* 1.6 ± 0.4 GPa and *E*_5%_ *=* 1.8 ± 0.1 GPa). Furthermore, we observed more than 100% difference between measurements carried out over different samples at the same temperature and degree of cross-linking. In Figure 6 we compare experimental stress measurements during elongation for a single sample to those of simulations, also calculated for a single sample. It is seen that while the computed measurements resemble the experimental trend, the fluctuation in the stress in the simulated system is extremely large due to the small sample size.

**Figure 7:**
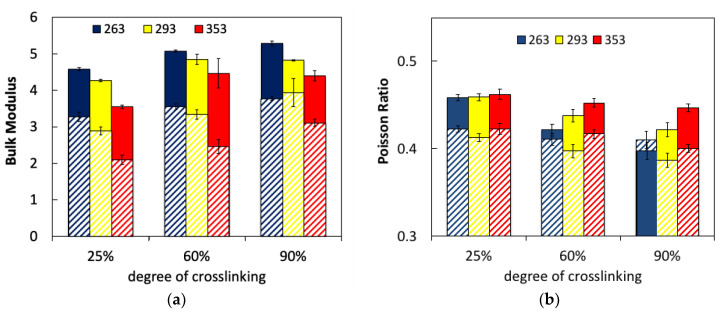
Molecular dynamics results of the effect of the temperature at three chosen degrees of crosslinking on the (**a**) bulk modulus calculated from compression at a rate of 10^7^ s^−1^ (striped bars) and fluctuations (solid bars) and (**b**) Poisson’s ratio calculated from Equation (2) based on tensile deformation rates of 10^7^ s^−1^ (solid bars) and 10^9^ s^−1^ (striped bars).

Assuming that the material is isotropic and within the elastic regime, then Poisson’s ratio should be bounded by two theoretical limits: −1.0 ≤ *ν* ≤ 0.5. Since the substance tends to become thinner in the transverse direction, it usually results in a positive Poisson’s ratio. Based on the experimental measurements (Table 1), *ν* for the epoxy should be ca. 0.39 and should increase substantially with temperature to 0.45 at the highest temperature tested. The increase of *ν* with temperature is due to the greater molecular motion that leads to an effectively softer material. However, Poisson’s ratio measured directly from the simulations (Figure 7) reveals little dependence on the temperature and the degree of crosslinking (as previously observed in the simulation of a similar system [17]), with average values around 0.46–0.6 (Figure 7b). The lack of internal symmetry in the small samples used in the simulation may explain the relatively large values obtained for *ν* in comparison with experiments, as no theoretical limit exists for anisotropic systems [28]. In addition to the inherent anisotropy present in small samples, it should also be noted that Poisson’s ratio calculated in this manner involves the division of two small numbers. The large statistical error inherent to the small sample size can therefore lead to large deviations in the calculated *ν*.

We define a measure of anisotropy, A, that varies between null (for isotropic material) and unity,

(1) $$\bar{A}=\langle \frac{E_{\max}-E_{\min}}{E_{\max}}\rangle$$

where the maximum and minimum values correspond to measurements of the elastic modulus over the three axial directions for each sample and the angular brackets denote an average over the five independent samples. We find that $\bar{A}$ decreases somewhat with the degree of crosslinking, though remains quite high with values of 0.42 ± 0.06, 0.40 ± 0.07, and 0.36 ± 0.02, for 25%, 60%, and 90% degree of crosslinking, respectively. This suggests that our samples are indeed highly anisotropic. Notably, $\bar{A}$ approaches unity for measurements sampled from 2% strain.

A better estimate of Poisson’s ratio was obtained from its relation to the elastic modulus and the bulk modulus, *K*, given in Equation (2):

(2) $$\nu =\frac{1}{2}-\frac{E}{6K}\;$$

A notable advantage of molecular simulations is the ease of calculation of the bulk modulus—a thermodynamic quantity that measures a material’s resistance to uniform externally applied pressure. Information about the dependence of the bulk modulus on temperature for epoxies is quite limited. The bulk modulus is inversely proportional to the compressibility and generally decreases with an increase in temperature above *T_g_*. *K* is proportional to the fluctuations in the volume of the sample at equilibrium, which increase with temperature as the polymer chains gain more mobility. *K* is less sensitive to change at temperatures below *T_g_*. Overall, the bulk modulus demonstrates a low sensitivity to crosslinking density, particularly at higher degrees of crosslinking. There is a maximum 25% difference between 50% degree of curing and a fully cured sample [29].

The bulk modulus can either be calculated from volume fluctuations in isothermal-isobaric simulation, or from non-equilibrium simulations of compression or expansion. While the former method requires lengthy simulations to minimize fluctuations, measurements using the latter method depend on the rate of compression or expansion. We calculated the bulk modulus using both methods. According to the fluctuation-dissipation theorem for a system at equilibrium, *K* can be calculated from volume fluctuations under uniform stress, as it is proportional to the inverse of the compressibility,

(3) $$\frac{\langle V^{2}\rangle -\langle V\rangle^{2}}{VkT}=\frac{1}{K}\;$$

where the brackets indicate ensemble average. For non-equilibrium compression measurements, the following relation is used,

(4) $$K=-V{\left(\frac{\partial p}{\partial V}\right)}_{p}=RT\frac{\langle V\rangle }{\langle {\left(\delta V\right)}^{2}\rangle }$$

where *δV* = *V −* 〈*V*〉. Average bulk moduli that were obtained from compression simulations and from fluctuations at equilibrium are compared in Figure 7a. The average values obtained are within the range reported in the literature for other epoxy systems from both experiments and simulations. As expected, the bulk modulus decreases with increasing temperature, and increases somewhat with the degree of crosslinking due to the additional local stress that is induced in the system. While the same trends for the change of *K* with temperature and the degree of crosslinking are observed for the two different methods of measurement, the differences are striking. On average, the calculations are 30% higher based on fluctuations. Dependence on the method of measurement has been observed previously with simulations [18]. The rate of compression was also found to have a moderate effect on the measured bulk modulus. Figure 7a shows that two orders of magnitude higher compression rates result only in a 15% increase in the modulus. However, high deformation rates produce results with more scatter and less consistent trends.

Poisson’s ratio predicted from measurements of the elastic and bulk moduli based on Equation (2) is shown in Figure 7b. Excellent agreement with our experimental measurements is observed at 90% degree of crosslinking and simulated deformation rate of 10^–7^ fs^–1^, ranging from 0.39 at 263 K to 0.45 at 353 K. We further observe that low degrees of crosslinking result in relatively high values of *ν* (~0.46) that are independent of temperature due to the liquidlike nature of the weakly crosslinked sample. Increasing the degree of crosslinking leads to a significant decrease in *ν*, particularly at lower temperatures. We note that the higher rates of deformation (of 10^−9^ fs^−1^) gave overall lower values of *ν* around 0.4. However, little sensitivity to the degree of crosslinking or temperature was observed. Higher deformation rates effectively correspond to material behavior at lower temperatures [24,30] where a lower Poisson’s ratio is expected.

**Figure 8:**
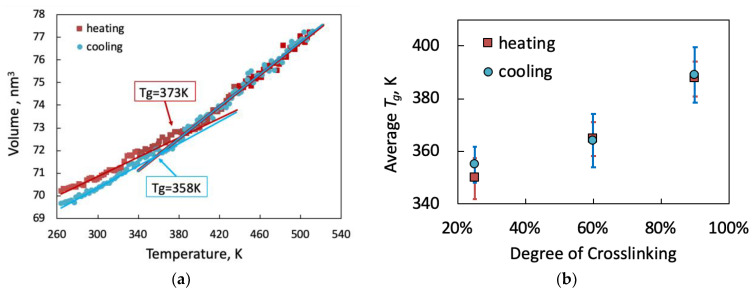
(**a**) Volume change during temperature increment and decrement around glass transition at 90% degree crosslinking and (**b**) average glass transition temperature as a function of degree of crosslinking evaluated during heating and cooling simulations.

The experimentally measured glass transition temperature for the system under consideration ranged between 43 and 60 °C, depending on the number of heating cycles and aging. Although the glass transition is not a discrete thermodynamic transition, but rather occurs over a range of temperatures, it is common to report one point within that range where the slope of volume versus temperature changes. The transition is continuous for the small samples studied in molecular simulation [31], as can be seen in Figure 8a. *T_g_* was, therefore, extracted from the crossing of initial slopes of the volume-temperature curves during both heating and cooling (at an effective rate of 25 K/ns). As with *E*, the range of data chosen for the calculation of *T_g_* can significantly affect its value.

The glass transition temperature is particularly sensitive to the heating or cooling rate, as enough time must be allowed for various molecular relaxation processes to take place. The slowest rates accessible to atomistic models are still 7–10 orders of magnitude higher than the fastest experimental rates. On the one hand, a much lower predicted *T_g_* might be anticipated for the low molecular weight polymer chains considered in the simulation [32]. On the other hand, the lower free volume present in systems of shorter chains [25] leads to reduced chain mobility and hence higher *T_g_*. However, the overwhelming effect is that of the short timescales for heating (or cooling), which leads to brittle behavior [30] and hence overprediction of *T_g_*. Under such conditions, sufficient chain relaxation cannot occur, and the rubbery state acts as a glassy state. Interestingly, Afzal et al. [33] found that the discrepancy between measured and calculated *T_g_* is consistent within a simulation protocol, and hence could be accounted for with proper calibration. Alternatively, the difference between experimental and computational rates could potentially be bridged using time-temperature superposition [24,31].

Interestingly, we observe a difference in *T_g_* measured from heating or from cooling of approximately 15 °C for a given sample, as shown in Figure 8a. However, the average *T_g_* calculated over the five independent crosslinked samples (Figure 8b) is much less sensitive to whether it is calculated from heating or from cooling, as well as to the choice of range of data for extracting the slope. The results obtained from the simulations are expectedly higher than experimental values (by 75–115 K), but in agreement with computational studies on similar systems [17]. Notably, while similar average values of *T_g_* are obtained from both heating and cooling simulations, the error in the calculation is consistently larger for *T_g_* extracted from cooling. Lower temperatures tend to require longer equilibration periods to allow for sufficient relaxation of the chains. As the temperature is reduced, the chains get trapped in locally hindered conformations and do not have sufficient time to relax.

The volumetric coefficient of thermal expansion $\alpha_{V}=\frac{1}{V}{\left(\frac{\partial V}{\partial T}\right)}_{p}$ can be extracted from the slopes of the volume-temperature plots in the glassy and rubbery regions before and after *T_g_*, respectively. It is expected that $\alpha_{V}$ will decrease with the increase of crosslinking degree as the overall stiffness of the matrix increases. The values of $\alpha_{V}$ that were obtained during heating or cooling are in the range of 20–33 × 10^−5^/°C for the glassy state. $\alpha_{V}$ values for the rubbery state were more sensitive to the procedure used. For heating, we obtained values in the range of 42–56 × 10^−5^/°C for the rubbery state, and 54–63 × 10^−5^/°C during cooling. In both methods, the values coincide with previous molecular simulation studies for a similar system [31], which tends to be consistently higher than experimental results reported in the literature, around 5–9 × 10^−5^/°C for the glassy state and 16–30 × 10^−5^/°C for the rubbery state [34].

## 5. Conclusions

Simulations of crosslinked epoxy resins may be used to cheaply and systematically investigate the relationship between chemistry and material properties. However, since the chemistry of industrial products is often complex, reported simulations have concentrated on idealized systems, with DEGBA/DETDA among the most popular. In this work, we modeled a 4-component epoxy often used with adhesive joints using the united-atom OPLS forcefield and compared its mechanical and thermomechanical properties with experimental measurements. The predicted elastic modulus shows fair agreement with experimental measurements over the range of temperatures studied. The simulated *E* is within 20% of the experimental value at 263 K, however the accuracy diminishes with increasing temperature, with a 70% discrepancy at 353 K. Moreover, the spread of *E* obtained from the simulation results is wide and can reach up to 80% error. Poisson’s ratio was found to be predicted accurately from the relation between *E*, *K*, and *ν*, but not from direct measurements from elastic deformation. The glass transition temperature is overpredicted by up to 50 °C as expected, due to the high cooling (or heating) rate. Throughout this work, we focused on the statistics of a small sample size and its implications towards prediction of mechanical properties of an industrial epoxy. We found that the variance in calculated properties measured for independently crosslinked samples is very large, though the trends with degree of crosslinking and temperature are qualitatively consistent with experiments. However, even for the complex chemistry in this study, simulations carried out on a small number of independent crosslinked samples improve the quantitative agreement between simulation and experiments, despite a large variance in calculated properties. 

## Figures and Tables

**Table 1:** Tensile properties of EPON 815C epoxy cured with Epikure^TM^ 3140.

	−5 °C	RT	60 °C
Modulus [GPa]	3.0	2.6	0.3
Tensile strength [MPa]	2.6	40	22
Poisson’s ratio	0.39	0.37	0.45
Elongation at failure [%]	0.7	1.7	1.6

**Table 2:** Glass transition temperature of EPON 815C/Epicure 3140 measured at room temperature.

	After 7 Days	After 1 Year
*T_g_* [°C] first heating cycle	43	57
*T_g_* [°C] second heating cycle	49	60

**Table 3:** Comparison of calculated density of epoxy constituents with manufacturer data at 25 °C.

Compound	Density [g/cm^3^]	Calculated Density [g/cm^3^]
DGEBA	1.13	1.156 ± 0.003
BGE	0.91	0.897 ± 0.004
Epikure 3140	0.97	0.965 ± 0.005
EPON	1.10	1.126 ± 0.008
Epoxy	1.12	1.117 ± 0.003

## Data Availability

Complete raw data from the molecular dynamics simulations are available from Mendeley Data, V1, doi: 10.17632/zxdxp326dd.1.
